# Skeletal muscle denervation investigations: selecting an experimental control wisely

**DOI:** 10.1152/ajpcell.00441.2018

**Published:** 2019-01-09

**Authors:** Haiming Liu, LaDora V. Thompson

**Affiliations:** ^1^Division of Gerontology and Geriatric Medicine, Department of Medicine, School of Medicine, University of Washington, and Geriatric Research, Education, and Clinical Center, Veterans Affairs Puget Sound Healthcare System, Seattle, Washington; ^2^Department of Physical Therapy and Athletic Training, Boston University, Boston, Massachusetts

**Keywords:** crossover effect, muscle atrophy, proteasome, proteolysis, tibial nerve transection

## Abstract

Unilateral denervation is widely used for studies investigating mechanisms of muscle atrophy. The “contralateral-innervated muscle” is a commonly used experimental control in denervation studies. It is not clear whether denervation unilaterally alters the proteolytic system in the contralateral-innervated muscles. Therefore, the objectives of this rapid report are *1*) to determine whether unilateral denervation has an effect on the proteolytic system in contralateral-innervated control muscles and *2*) to identify the changes in proteasome properties in denervated muscles after 7- and 14-day tibial nerve transection with either the contralateral-innervated muscles or intact muscles from nonsurgical mice used as the experimental control. In the contralateral-innervated muscles after 7 and 14 days of nerve transection, the proteasome activities and content are significantly increased compared with muscles from nonsurgical mice. When the nonsurgical mice are used as the experimental control, a robust increase in proteasome properties is found in the denervated muscles. This robust increase in proteasome properties is eliminated when the contralateral-innervated muscles are the experimental control. In conclusion, there is a crossover effect from unilateral denervation on proteolytic parameters. As a result, the crossover effect on contralateral-innervated muscles must be considered when an experimental control is selected in a denervation study.

## INTRODUCTION

Denervation is a widely used experimental model to elucidate the mechanisms underlying muscle atrophy. Upon denervation, a loss of muscle mass is noted as early as 7 days, and this rapid loss is primarily due to the degradation of myofibrillar proteins. The ubiquitin-proteasome system is responsible for degradation of the myofibrillar proteins, yet the magnitude of the denervation-induced response of the proteasome properties in the denervated muscles is highly variable (5, 9, 10, 16, 17).

A critical analysis of the published studies investigating the effects of denervation reveals the use of the contralateral-intact limb or “contralateral-innervated muscle” of the mouse, instead of a separate control group of mice without a denervation procedure, as the experimental control (3, 5, 9, 15, 16, 17). Although the use of the contralateral-innervated muscle as the experimental control is customary, this practice may underlie the variable denervation-induced responses. Furthermore, this practice may influence the results. Whether the surgical procedure of nerve transection alters the characteristics of the ubiquitin-proteasome system in the contralateral-innervated muscles is unknown.

Therefore, this rapid report has two purposes: *1*) to determine whether denervation has an effect on proteolysis in the contralateral-innervated control muscle and *2*) to identify which experimental control, the contralateral-innervated muscle of a mouse with a nerve transection or the innervated muscle from a nonsurgical mouse, best serves to detect denervation-induced proteasome characteristics. We hypothesize that the proteasome properties in the contralateral-innervated muscles will be increased in the mice receiving the denervation surgery. This hypothesis is based on reported crossover effects under conditions of exercise, electrical stimulation, inflammation, and injury (2, 6, 19, 23). We further hypothesize that the denervation response of the properties of proteolysis will be attenuated when the contralateral-innervated muscle is designated the control group compared with when an intact muscle from a nonsurgical mouse is the control group.

## METHODS

### 

#### Experimental design.

There were four experimental groups: external control (Ex Ctrl), 7-day-denervated (7d DN), 14-day-denervated (14d DN), and sham (Sham Ctrl). The mice in the Ex Ctrl group did not undergo surgery, and the gastrocnemius (GAS) muscles were innervated and identified as intact (innervated muscle from a nonsurgical mouse). In contrast, the mice in the denervated groups (7d DN and 14d DN) underwent tibial nerve transection surgery on the left hindlimb and were allowed to recover for 7–14 days. The mice in the Sham Ctrl group underwent the same surgery as the mice in the denervated groups; however, the tibial nerve was not transected.

**Fig. 1.**
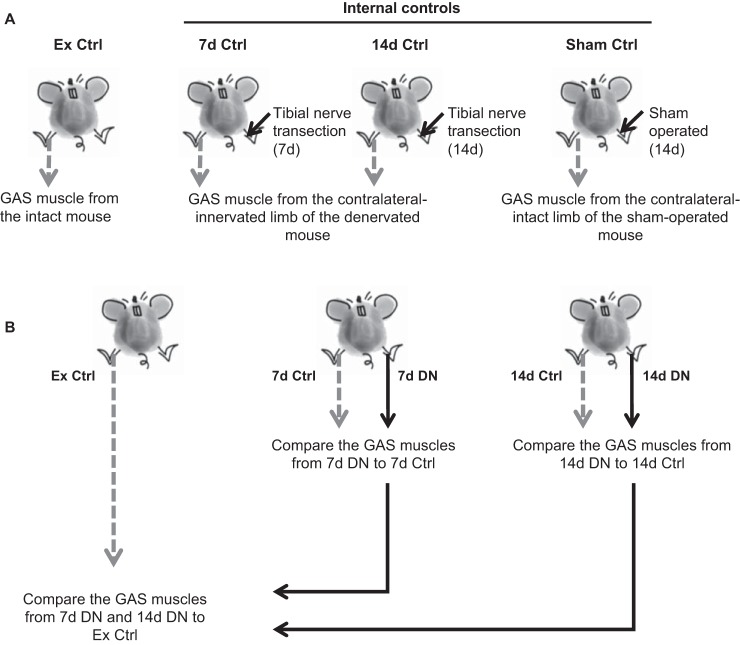
Experimental design. *A*: proteasome properties in control groups. Proteasome properties were detected in gastrocnemius (GAS) muscle from the intact [external control (Ex Ctrl)] mouse, contralateral-innervated limb of the 7-day-denervated (7d Ctrl) and 14-day-denervated (14d Ctrl) mice, and contralateral-intact limb of the sham-operated (Sham Ctrl) mouse. *B*: dependence of denervation-induced changes in proteasome properties on the “control” used for comparison. Proteasome properties in 7-day denervated (7d DN) and 14-day denervated (14d DN) GAS muscles were compared with Ex Ctrl separately (*left*); then these parameters from 7d DN and 14d DN were compared with 7d Ctrl and 14d Ctrl, respectively (*right*).

To evaluate the influence of the surgery (with or without the nerve transection) between the muscles identified as experimental controls, we compared the GAS muscle proteasome properties between the Ex Ctrl group and the internal control (7d Ctrl, 14d Ctrl, and Sham Ctrl) groups (Fig. 1*A*). Specifically, the GAS muscles from the intact nonsurgical mice are defined as Ex Ctrl. The GAS muscles from three “internal control” groups are as follows: the contralateral-innervated GAS muscles from the mice that underwent 7 and 14 days of denervation (7d DN and 14D DN) and the intact GAS muscles from the sham-operated (Sham Ctrl) mice.

To determine which experimental control group (external or internal) best serves to identify the effect of denervation on proteasome properties, we compared the proteasome properties from 7d DN or 14d DN muscles with either the Ex Ctrl or the internal controls (7d Ctrl or 14d Ctrl) (Fig. 1*B*).

#### Animals.

Male 5- to 7-mo-old C57BL/6 mice were randomly assigned to one of four experimental groups. All mice were housed under the same conditions and fed ad libitum. At the end point, GAS muscles from both hindlimbs were collected, flash-frozen in liquid nitrogen, and stored in a freezer at −80°C for future use. All procedures were approved by the University of Minnesota Institutional Animal Care and Use Committee (protocol no. 1306-30685A).

#### Tibial nerve transection.

The transection procedure is described elsewhere (4, 13). Briefly, mice in the denervation groups were anesthetized using isoflurane, and a 3-mm piece of the tibial nerve was removed. The sham mice underwent the same procedure without removal of the tibial nerve piece.

#### Enriched proteasome preparation and activity assay.

The preparation of enriched proteasomes and the assay for proteasome activity are described elsewhere (7, 13). Protein concentration was determined by bicinchoninic acid assay (Thermo Scientific). The fluorogenic peptides LLE-7-amido-4-methylcoumarin (AMC) and LLVY-AMC (EMD Millipore) and VGR-AMC (Enzo Life Sciences) were used to detect caspase-, chymotrypsin-, and trypsin-like activities, respectively. AMC (Sigma-Aldrich) was used as the standard. Fluorescence assay was measured at 37°C in a multimode microplate reader (Synergy HTX, BioTek).

#### Western blotting.

The protein content of proteasome subunits was detected as previously described (7, 13). The enriched proteasome preparation was loaded onto a 13% SDS gel (4–30 μg/well) and then transferred to a polyvinylidene difluoride membrane. The membranes were blocked (5% nonfat dry milk in Tris-buffered saline-Tween 20) at room temperature for 1 h and then incubated with the primary antibodies anti-α_7_ (1:1,000 dilution; catalog no. PW8110), anti-multicatalytic endopeptidase complex-like 1 (MECL1, 1:500 and 1:1,000 dilutions; catalog no. PW8150), anti-low-molecular-mass protein 7 (LMP7, 1:1,000 dilution; catalog no. PW8355), anti-aryl hydrocarbon receptor 1 (Rpt1, 1:1,000 dilution; catalog no. PW9400), and anti-proteasome activator 28α (PA28α, 1:1,000 dilution; catalog no. PW8185) from Enzo Life Sciences and anti-β_1_ (1:1,000 dilution; catalog no. PA5-78135) and anti-β_5_ (1:1,000 dilution; catalog no. PA1-977) antibodies from Thermo Scientific overnight at 4°C. The corresponding secondary antibodies were applied at dilution of 1:10,000 to 1:16,000, depending on the primary antibody. The membranes were developed using SuperSignal West Dura Extended Duration chemiluminescence substrate (Pierce) and imaged using ChemiDoc XRS (Bio-Rad). The final protein content of each individual sample was expressed as a ratio to a blot control (4, 7, 13).

#### Statistics.

Dunnett’s *t*-tests were conducted to detect the difference between each control group (7d Ctrl, 14d Ctrl, and Sham Ctrl) and the Ex Ctrl group (*P* < 0.05) and between each denervated group (7d Ctrl and 14d Ctrl) and the Ex Ctrl group (*P* < 0.05). Paired *t*-tests were performed to compare the differences between the denervated muscles and their contralateral internal controls (7d DN vs. 7d Ctrl and 14d DN vs. 14d Ctrl, *P* < 0.05). IBM SPSS version 22 was used for all comparisons. Values are means ± SE.

## RESULTS

### 

#### Comparison between experimental controls.

**Fig. 2.**
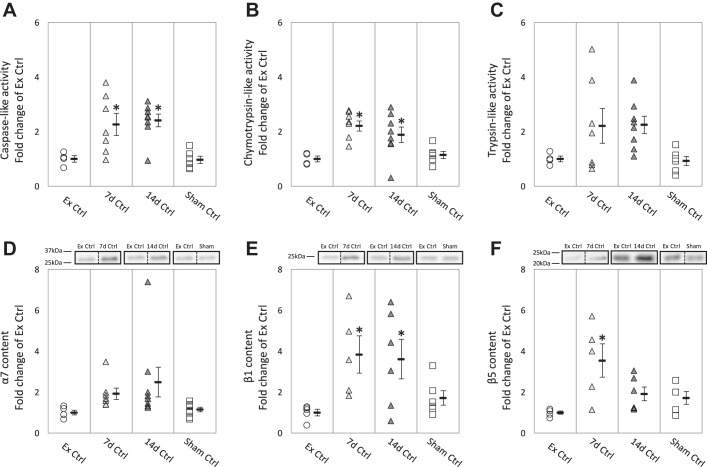
Proteasome properties in gastrocnemius muscles from internal controls (7d Ctrl, 14d Ctrl, and Sham Ctrl) and external control (Ex Ctrl). Data points represent fold change of Ex Ctrl from individual mice. *D–F*: representative Western blots for each corresponding proteasome subunit. Images of representative bands in each panel were from the same blot; dashed lines indicate rearrangement of the noncontinuous lanes. Values are means ± SE; *n* = 5, 7, 8, and 8 for α_7_ and *n* = 5, 5, 6, and 6 for the other proteasome properties in Ex Ctrl, 7d Ctrl, 14d Ctrl, and Sham Ctrl, respectively *P < 0.05 vs. Ex Ctrl.

We first set out to determine the effect of tibial nerve transection on the proteasome activities and content of the experimental control groups. Briefly, the main site for proteolysis is the 26S proteasome, which consists of the 20S catalytic core and the 19S regulatory complex. The outer α-subunits of the 20S core interact with the regulatory complex to translocate the ubiquitinated protein substrates into the core. Then the inner three pairs of catalytic β-subunits (β_1_, β_2_, and β_5_) carry out caspase-, trypsin- and chymotrypsin-like activities, to cleave after acidic, basic, and hydrophobic amino acids, respectively. We found significant differences in proteasome activities and content between our designated control groups, demonstrating a denervation-induced crossover response. Specifically, the nerve transection influenced proteolysis within the intact muscles of the contralateral limb (Fig. 2).

#### Proteasome activities.

Proteasome activities were greater in the contralateral-innervated GAS muscles (intact) at 7 and 14 days of denervation than the Ex Ctrl (Fig. 2, *A*–*C*). Although no significant difference was detected in trypsin-like activity in 7d Ctrl and 14d Ctrl, the individual data showed a pattern of increase (Fig. 2*C*). Caspase- and chymotrypsin-like activities of 7d Ctrl increased by 1.2- to –1.3-fold compared with Ex Ctrl (Fig. 2, *A* and *B*). Similarly, 14d Ctrl exhibited increases in caspase-like (1.4-fold) and chymotrypsin-like (0.9-fold) activities (Fig. 2, *A* and *B*). Figure 2 highlights the dispersion or spread of the proteasome activities in the internal control groups.

To identify whether the difference in proteasome activities between internal controls and Ex Ctrl is due to the incision surgery or severing of the nerve, we analyzed the proteasome activities from Sham Ctrl, a group of mice with only skin and muscle incision on the contralateral leg (Fig. 1*A*). There was no significant difference in the proteasome activities between Ex Ctrl and Sham Ctrl (Fig. 2), which indicated that the elevated proteasome activities in the internal controls were associated with the nerve being severed on the contralateral side, rather than the incision surgery.

#### Proteasome content.

Consistent with the proteasome activities, the subunit content demonstrated a crossover effect associated with unilateral tibial nerve transection. Figure 2, *E* and *F*, shows the one- to threefold increases in β_1_ and β_5_ in 7d DN and/or 14d DN in the contralateral-innervated muscles. It is likely that the augmented β_1_ and β_5_ content contributes to the increase in their corresponding activities: caspase- and chymotrypsin-like activities, respectively. Although no significant difference was found across the groups in α_7_ content, the individual data showed a pattern of increase. Similarly, the inducible forms of the standard proteasome (immunoproteasome subunits LMP7 and MECL-1), which play a role in influencing proteasome activities, also increased in the contralateral-innervated muscles compared with the Ex Ctrl (Table 1). However, the regulatory complex subunits Rpt1 and PA28α were not different (Table 1). As expected, there was no significant difference in the aforementioned subunits between Ex Ctrl and Sham Ctrl (Fig. 2, Table 1).

**Table 1.** Comparisons of other proteasome properties in gastrocnemius muscle

		Controls					
		Internal				Denervated	
Proteasome Properties	Measures	7d Ctrl	14d Ctrl	Sham Ctrl	Ex Ctrl	7d DN	14d DN
Immunoproteasome subunits	LMP7	0.9 ± 0.1 (5)	1.1 ± 0.1* (6)	0.6 ± 0.1 (6)	0.7 ± 0.0 (5)	1.4 ± 0.2*† (5)	1.3 ± 0.1* (6)
	MECL-1	1.5 ± 0.1* (5)	1.5 ± 0.1* (6)	0.7 ± 0.1 (6)	0.8 ± 0.0 (5)	2.0 ± 0.2* (5)	1.7 ± 0.1* (6)
19S regulator	Rpt1	2.5 ± 0.7 (7)	1.6 ± 0.4 (7)	1.0 ± 0.1 (6)	1.3 ± 0.2 (5)	6.4 ± 1.8* (7)	3.4 ± 0.6 (6)
11S regulator	PA28α	0.8 ± 0.1 (5)	1.3 ± 0.2 (6)	0.9 ± 0.1 (6)	0.9 ± 0.1 (5)	0.9 ± 0.1 (5)	1.9 ± 0.2* (6)

Values are means ± SE (arbitrary units); no. of mice shown in parentheses. Other proteasome properties were measured in gastrocnemius muscles from internal controls (7d Ctrl and 14d Ctrl), sham control (Sham Ctrl), external control (Ex Ctrl), and denervated (7d DN and 14d DN) groups. LMP7, low-molecular-mass protein 7; MECL-1, multicatalytic endopeptidase complex-like 1; Rpt1, aryl hydrocarbon receptor 1; PA28α, proteasome activator 28α.

* *P* ≤ 0.05 vs. Ex Ctrl.

† *P* < 0.05 vs. 7d Ctrl; no significant difference was detected between 14d DN and 14d Ctrl.

#### Denervation and the “control group”: which control group is the best?

Because we found that proteasome activities in the contralateral-intact muscles were increased after nerve transection, it is possible that the practice of using the contralateral-intact muscles as the experimental control may impact the research results. To determine the impact of this crossover effect, we compared the denervated muscle properties with the Ex Ctrl and the internal control groups (Fig. 1*B*). Notably, we found robust denervation-induced responses in the proteasome activities when the Ex Ctrl group was used, and these robust responses were eliminated when the contralateral-internal control group was used.

**Fig. 3.**
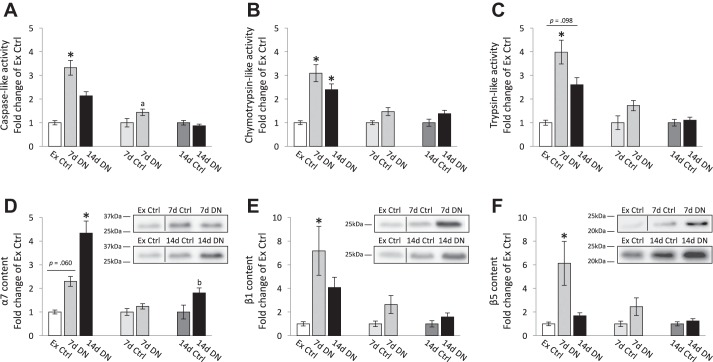
Proteasome properties of 7- and 14-day denervated muscles (7d DN and 14 DN) and designated control muscles [external control (Ex Ctrl) or internal controls (7d Ctrl/14d Ctrl)]. Results are presented as fold change of designated control. *D–* *F*: representative Western blots for each corresponding proteasome subunit. Images of representative bands in each panel were from the same blot; dashed lines indicate rearrangement of the noncontinuous lanes. Values are means ± SE; *n* = 5, 7, 7, 8, and 8 for α_7_ and *n* = 5, 5, 5, 6, and 6 for the other proteasome properties in Ex Ctrl, 7d Ctrl, 7d DN, 14d Ctrl, and 14d DN, respectively. *P ≤ 0.05 vs. Ex Ctrl. ^a,b^*P* < 0.05 vs. internal controls.

The caspase-like activity increased robustly (2.0-fold) in 7d DN compared with Ex Ctrl (Fig. 3*A*). However, the increase in 7d DN was <1.0-fold compared with its internal control. No significant difference was detected in caspase-like activity between 14d DN and either the Ex Ctrl or its internal control.

Chymotrypsin- and trypsin-like activities showed similar responses: statistical 1.4- to 3.0-fold increase in 7d DN and 14d DN compared with Ex Ctrl (Fig. 3, *B* and *C*; trend in Ex Ctrl vs. 14d DN in trypsin-like activity). In contrast, no change in chymotrypsin- and trypsin-like activities was detected between 7d DN and 14d DN and their internal controls (7d Ctrl and 14d Ctrl). The results of these comparisons strongly support the inclusion of an external experimental control group in the design of unilateral nerve transection experiments.

The effect of denervation on the proteasome subunit content (α_7_, β_1_, and β_5_) in the denervated GAS muscles was identified and compared with either the Ex Ctrl or their internal controls (7d Ctrl or 14d Ctrl). Similar to the results found with the proteasome activities, robust changes were observed in the content of the proteasome subunits from the 20S core in the denervated muscles compared with the Ex Ctrl vs. the internal controls. As shown in Fig. 3*D*, α_7_ content increased 1.0-fold in 7d DN (trend) compared with Ex Ctrl. This increase was eliminated when 7d DN was compared with the internal control (7d DN vs. 7d Ctrl). A significant increase in α_7_ content was observed in 14d DN: 3.0-fold compared with Ex Ctrl but only 1.0-fold compared with the internal control.

Significant increases in β_1_ and β_5_ content (5.0- to 6.0-fold) were detected in 7d DN compared with Ex Ctrl but not compared with their respective internal controls (Fig. 3, *E* and *F*). No significant difference was found in either β_1_ or β_5_ content in 14d DN compared with any of the control groups. Significant increases in immunoproteasome (LMP7 and MECL-1) and regulatory subunits (Rpt1 and PA28α) were induced by 7d DN and 14d DN compared with Ex Ctrl. Compared with the internal control, however, the responses were partially or totally eliminated, depending on the subunit and the duration of the denervation (Table 1). Consistent with the proteasome activities, the results of the proteasome subunit content strongly support the inclusion of an external experimental control group in the design of unilateral nerve transection experiments.

## DISCUSSION

Collectively, we found a crossover response in the proteasome properties in the contralateral-intact muscles following the unilateral tibial nerve transection. The sham treatment did not trigger a crossover response in the contralateral-intact muscles. This finding clearly shows that the effect of denervation on the contralateral-innervated muscles was primarily from the nerve transection, rather than other factors during surgery. Evidence for the contralateral crossover response is present under several conditions, such as exercise, fatigue, and massage, with both positive and negative effects (6, 8, 14, 20, 23).

The physiological mechanisms that mediate the contralateral denervation effects are likely neural. Koltzenburg first suggested that neural mechanisms were responsible for the crossover response from a peripheral nerve transection primarily through the spinal cord (12). In this crossover mechanism, the damaged peripheral neurons affect the contralateral side by altering the neuronal activity (sympathetic nervous system) directly or by producing changes in trophic factors [e.g., brain-derived neurotrophic factor and neurotrophin 3 (18)] that interrupt the intact neuromuscular junctions of the contralateral muscle (11). In contrast to the direct neural mechanisms through the spinal cord, elevated systemic mediators induced by the specific nerve damage, such as immune cells and stress-related hormones, may contribute to the crossover effect. For instance, sciatic nerve crush/transection induced increases in the serum immunoglobulins after 7 and 14 days (22) and by the release of myokines from the denervated muscle that influences other muscles at distant sites, which has been shown with exercise (1). There is no direct evidence that the crossover response is from systemic mediators. Because the responses in the contralateral intact muscles are widespread, these mechanisms may not be mutually exclusive, and it is possible that many mechanisms are involved in the response.

Most often, the approach in denervation studies is to utilize the contralateral limb as an internal control for reasons such as animal number and cost-effectiveness. Among these denervation studies using the contralateral limb as an internal control, only a few investigated proteasome characteristics in response to more severe denervation methods: sciatic nerve transection or crush (9, 16, 21). Interpretation of these studies is difficult because of the inconsistent findings. For instance, there is no change in proteasome activities in tibialis anterior muscles (16), whereas a 1.0-fold increase is reported in soleus (9) and GAS/plantaris (21) muscles after 7 days of denervation. Although the denervation methods and the muscles investigated are different, the evidence is clear: the denervation-induced changes in proteasome properties are less robust than the results reported in the current study, which were compared with an independent external control group. The report of no change in proteasome properties (16) is consistent in the current study when the contralateral limb is used as the internal control.

In summary, unilateral tibial nerve transection for 7 or 14 days induces a crossover effect in the contralateral-innervated muscles, increasing the activities of the proteasome and the content of the proteasome subunits. This crossover denervation response in the contralateral-innervated muscles impacts the results when the contralateral-innervated muscles are used as experimental controls in the research design. The tibial nerve transection results in increases in proteasome characteristics in the denervated muscles when the experiments use nonsurgical mice as the designated controls. These robust increases in proteasome characteristics following nerve transection are eliminated when the study is designed to use the contralateral-innervated muscles of the mice undergoing the tibial nerve transection. The best available conclusion from the current study is that a higher degree of experimental control is necessary in the design of denervation studies. Future studies are required to investigate underlying mechanisms responsible for the proposed crossover effect.

## GRANTS

This work was supported by National Institute on Aging Grants T32 AG-029796 and R01AG-017768, a Travis M. Roy Endowed Professorship, a Doctoral Dissertation Fellowship (University of Minnesota, 100007249), and Department of Veterans Affairs MERIT Grant I01-BX000507.

## DISCLOSURES

No conflicts of interest, financial or otherwise, are declared by the authors.

## AUTHOR CONTRIBUTIONS

H.L. and L.V.T. conceived and designed research; H.L. performed experiments; H.L. analyzed data; H.L. and L.V.T. interpreted results of experiments; H.L. prepared figures; H.L. and L.V.T. drafted manuscript; H.L. and L.V.T. edited and revised manuscript; H.L. and L.V.T. approved final version of manuscript.
